# Clinical Implications of Discordant Early Molecular Responses in CML Patients Treated with Imatinib

**DOI:** 10.3390/ijms20092226

**Published:** 2019-05-06

**Authors:** Stefania Stella, Valentina Zammit, Silvia Rita Vitale, Maria Stella Pennisi, Michele Massimino, Elena Tirrò, Stefano Forte, Antonio Spitaleri, Agostino Antolino, Sergio Siragusa, Vincenzo Accurso, Donato Mannina, Stefana Impera, Caterina Musolino, Sabina Russo, Alessandra Malato, Giuseppe Mineo, Maurizio Musso, Ferdinando Porretto, Bruno Martino, Francesco Di Raimondo, Livia Manzella, Paolo Vigneri, Fabio Stagno

**Affiliations:** 1 Department of Clinical and Experimental Medicine, University of Catania, 95123 Catania, Italy; stefania.stel@gmail.com (S.S.); silviarita.vitale@gmail.com (S.R.V.); perny76@gmail.com (M.S.P.); michedot@yahoo.it (M.M.); ele_tir@yahoo.it (E.T.); manzella@unict.it (L.M.); vigneripaolo@gmail.com (P.V.); 2 Center of Experimental Oncology and Hematology, A.O.U. Policlinico Vittorio Emanuele, 95123 Catania, Italy; 3 Division of Hematology and Bone Marrow Transplant, AOU Policlinico - V. Emanuele, 95123 Catania, Italy; valentina.zammit@tiscali.it (V.Z.); dottspitaleriantonio@gmail.com (A.S.); diraimon@unict.it (F.D.R.); 4 Mediterranean Institute of Oncology, 95029 Viagrande, Italy; stefano.forte@grupposamed.com; 5 Department of Transfusional Medicine, Maria Paternò-Arezzo Hospital, 97100 Ragusa, Italy; agostino.antolino@asp.rg.it; 6 Division of Hematology, A.O.U. Policlinico “P. Giaccone”, University of Palermo, 90127 Palermo, Italy; sergio.siragusa@unipa.it (S.S.); casteldaccia@tiscali.it (V.A.); 7 Division of Hematology, Papardo Hospital, 98158 Messina, Italy; donamanni@gmail.com; 8 Division of Oncology and Hematology, ARNAS Garibaldi-Nesima, 95122 Catania, Italy; st.impera@alice.it; 9 Division of Hematology, University of Messina, 98125 Messina, Italy; cmusolino@unime.it (C.M.); sabinarusso@tiscali.it (S.R.); 10 Division of Hematology and Bone Marrow Transplant, Villa Sofia-Cervello Hospital, 90146 Palermo, Italy; alessandramalato@hotmail.com; 11 Division of Hematology, San Vincenzo Hospital, 98039 Taormina, Italy; giuseppe.mineo65@tiscali.it; 12 Division of Hematology, La Maddalena Hospital, 90146 Palermo, Italy; mamusso@libero.it (M.M.); fporretto@alice.it (F.P.); 13 Hematology Department, Grande Ospedale Metropolitano, Reggio Calabria, 89124 Reggio Calabria, Italy; brunmartin@libero.it; 14 Department of Surgery, Medical and Surgical Specialities, University of Catania, 95123 Catania, Italy

**Keywords:** chronic myeloid leukemia, *BCR-ABL1*, imatinib mesylate, European Leukemia Net, early molecular response

## Abstract

A reduction in *BCR-ABL1/ABL1^IS^* transcript levels to <10% after 3 months or <1% after 6 months of tyrosine kinase inhibitor therapy are associated with superior clinical outcomes in chronic myeloid leukemia (CML) patients. In this study, we investigated the reliability of multiple *BCR-ABL1* thresholds in predicting treatment outcomes for 184 subjects diagnosed with CML and treated with standard-dose imatinib mesylate (IM). With a median follow-up of 61 months, patients with concordant *BCR-ABL1/ABL1^IS^* transcripts below the defined thresholds (10% at 3 months and 1% at 6 months) displayed significantly superior rates of event-free survival (86.1% vs. 26.6%) and deep molecular response (≥ MR^4^; 71.5% vs. 16.1%) compared to individuals with *BCR-ABL1/ABL1^IS^* levels above these defined thresholds. We then analyzed the outcomes of subjects displaying discordant molecular transcripts at 3- and 6-month time points. Among these patients, those with *BCR-ABL1/ABL1^IS^* values >10% at 3 months but <1% at 6 months fared significantly better than individuals with *BCR-ABL1/ABL1^IS^* <10% at 3 months but >1% at 6 months (event-free survival 68.2% vs. 32.7%; *p* < 0.001). Likewise, subjects with *BCR-ABL1/ABL1*^IS^ at 3 months >10% but <1% at 6 months showed a higher cumulative incidence of MR^4^ compared to patients with *BCR-ABL1/ABL1^IS^* <10% at 3 months but >1% at 6 months (75% vs. 18.2%; *p* < 0.001). Finally, lower *BCR-ABL1/GUS^IS^* transcripts at diagnosis were associated with *BCR-ABL1/ABL1^IS^* values <1% at 6 months (*p* < 0.001). Our data suggest that when assessing early molecular responses to therapy, the 6-month *BCR-ABL1/ABL1^IS^* level displays a superior prognostic value compared to the 3-month measurement in patients with discordant oncogenic transcripts at these two pivotal time points.

## 1. Introduction

Chronic myeloid leukemia (CML) is characterized by a unique cytogenetic marker, the Philadelphia (Ph) chromosome, arising from the reciprocal translocation between the long arms of chromosomes 9 and 22 [1]. In turn, the Ph chromosome generates the *BCR-ABL1* fusion chimeric gene, encoding an oncoprotein with constitutive tyrosine kinase activity that alters the proliferation rate, survival signaling, immunological interactions, and cytoskeletal dynamics of the hematopoietic stem cell [2,3,4,5,6,7]. The introduction of the tyrosine kinase inhibitor (TKI) imatinib mesylate (IM) has radically improved the outcome of chronic phase CML patients by generating unprecedented rates of complete hematological (CHR) and cytogenetic (CCyR) responses and deep molecular responses (MR) [8,9]. Despite these excellent results, IM resistance is often detected in patients failing to achieve an optimal response (OR) as defined by the current European Leukemia Net (ELN) recommendations [10]. IM resistance includes both BCR-ABL1-dependent [11,12,13] and BCR-ABL1-independent mechanisms [14,15] that may be prevented or overcome by second- (2G) or third-generation (3G) TKIs such as dasatinib (DAS), nilotinib (NIL), bosutinib (BOS) and ponatinib (PON). Moreover, non-ABL1-directed inhibitors and immunological-targeting approaches are currently being developed as additional treatment strategies for the disease [16,17,18].

With the introduction of 2G and 3G TKIs, the early identification of CML patients at high risk of failing IM treatment has become of pivotal importance. Hence, several clinical prognostic scores [Sokal and EUTOS long-term survival (ELTS)] have been employed to predict CML response to IM at the time of diagnosis, thereby recognizing patients that will display inferior overall survival (OS) rates [19,20,21,22]. At the same time, several groups have begun to investigate early molecular parameters that might distinguish CML patients unlikely to benefit from IM. A seminal paper by Marin et al. reported that *BCR-ABL1/ABL1* transcript thresholds of 10% at 3 months and 1% at 6 months strongly predict long-term outcomes for CML patients [23]. Subsequently, Hanfstein and colleagues reported that *BCR-ABL1/ABL1^IS^* levels >1% at 6 months were associated with inferior 5-year OS compared to values <1%, thereby suggesting that *BCR-ABL1* transcripts at 6 months predict the response of CML to IM [24]. This body of evidence has been gradually incorporated into clinical practice. Currently, both the National Comprehensive Cancer Network (NCCN) and ELN guidelines include the failure to achieve selected molecular responses, albeit at different time points, as a reason to switch to a different TKI.

In this complex clinical and molecular scenario, a challenging issue is how to manage patients who display discordant *BCR-ABL1/ABL1^IS^* transcripts (*BCR-ABL1/ABL1^IS^* <10% at 3 months but >1% at 6 months or *BCR-ABL1/ABL1^IS^* >10% at 3 months but <1% at 6 months) at the 3- and 6-month time points. In this study, we investigated the clinical implications of these molecular landmarks, in subjects with discordant transcripts at the 3- and 6-month time points, in order to translate these molecular data into clinically meaningful information for CML patients receiving IM as first-line treatment for their disease.

## 2. Results

### 2.1. Patient Responses and ELN Outcomes

Patient characteristics are summarized in Table 1. Every patient achieved a CHR, 157 (85.3%) attained a CCyR, 143 (77.7%) reached a major molecular response (MR^3^) (median time 10 months; range, 3–83), and 90 (48.9%) achieved a deep molecular response (MR^4^) (median time 20.5 months; range, 6–83). Median follow-up of the accrued population was 61 months (range, 12–90). According to the 2013 ELN recommendations, 126 (68.5%) patients achieved an optimal response, 39 (21.2%) failed IM, 10 (5.4%) were classified as “warning”, and 9 (4.9%) discontinued IM due to drug intolerance. All individuals who discontinued IM because of drug failure or intolerance received 2G TKIs.

### 2.2. Probability of Event-Free Survival and Molecular Response According to BCR-ABL1/ABL1^IS^ Transcripts at 3 and 6 Months

**Figure 1:**
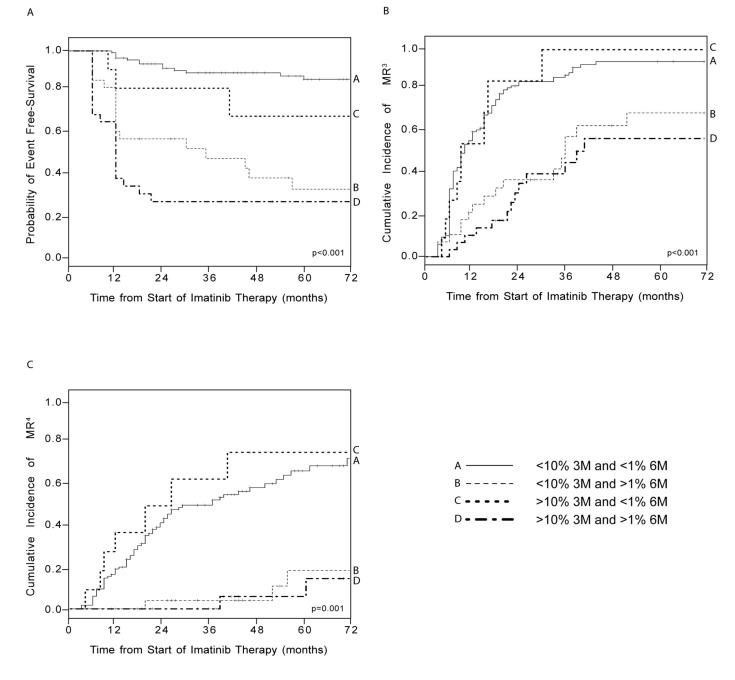
Event-free survival (EFS) estimates (**A**) and cumulative incidence of major molecular response (MR^3^) (**B**) and deep molecular response (MR^4^) (**C**) according to *BCR*-*ABL1/ABL1^IS^* transcripts at 3 and 6 months. Patients were divided into four groups according to their *BCR*-*ABL1* expression at the 3 and 6 months. EFS probability (**A**) and cumulative incidence of MR^3^ (**B**) and MR^4^ (**C**) were calculated for each group. Vertical lines indicate censored patients. *p*-values refer to statistical significance among all four groups included in the analyses.

Consolidated data has established that *BCR*-*ABL1*/*ABL1^IS^* levels of 10% at 3 months and 1% at 6 months are predictive of OS, progression-free survival (PFS), event-free survival (EFS), and CCyR [23,24]. When we stratified patients according to their *BCR-ABL1/ABL1^IS^* levels at 3 and 6 months, we found that 63% of the patients had concordant low transcripts (i.e., <10% at 3 months and <1% at 6 months, group A) while 15.8% of individuals displayed concordant high transcripts (i.e., >10% at 3 months and >1% at months, group D). EFS probability was 86.1% vs. 26.6% (*p* < 0.001) in patients respectively displaying concordant low values (group A) or concordant high values (group D) (Figure 1A).

The remaining 21.2% of patients displayed discordant transcript values at the 3- and 6-month time points, as 15.2% presented low (<10%) *BCR-ABL1/ABL1^IS^* at 3 months but high (>1%) levels at 6 months (group B), and 6% displayed high (>10%) *BCR-ABL1/ABL1^IS^* at 3 months, but low (<1%) levels at 6 months (group C). Among subjects with discordant *BCR-ABL1* expression, group B (<10% at 3 months but >1% at 6 months) achieved significantly lower 6-year EFS as compared to group C (>10% at 3 months but <1% at 6 months) (32.7% vs. 68.2%, *p* < 0.001; Figure 1A).

We next evaluated if the 3- or the 6-month *BCR-ABL1/ABL1^IS^* levels would predict subsequent molecular responses to IM. Therefore, we compared the cumulative incidence of MR^3^ and MR^4^ in the four groups described above. As expected, there were significant differences between groups A and D. Specifically, patients with low transcripts at both 3 and 6 months showed significantly higher cumulative incidences of both MR^3^ (94.2% for group A vs. 57.1% for group D, *p* < 0.001; Figure 1B) and MR^4^ (71.5% for group A vs. 16.1% for group D, *p* < 0.001; Figure 1C). As for patients with discordant BCR-ABL1/ABL1^IS^ levels, individuals in group B displayed significantly lower cumulative incidences of both MR^3^ (69.4% vs. 100% for group C, *p* < 0.001; Figure 1B) and MR^4^ (18.2% vs. 75% for group C, *p* < 0.001; Figure 1C).

### 2.3. Correlation between BCR-ABL1/GUS^IS^ Levels at Diagnosis and BCR-ABL1/ABL1^IS^ Transcripts at 3 and 6 Months

**Figure 2:**
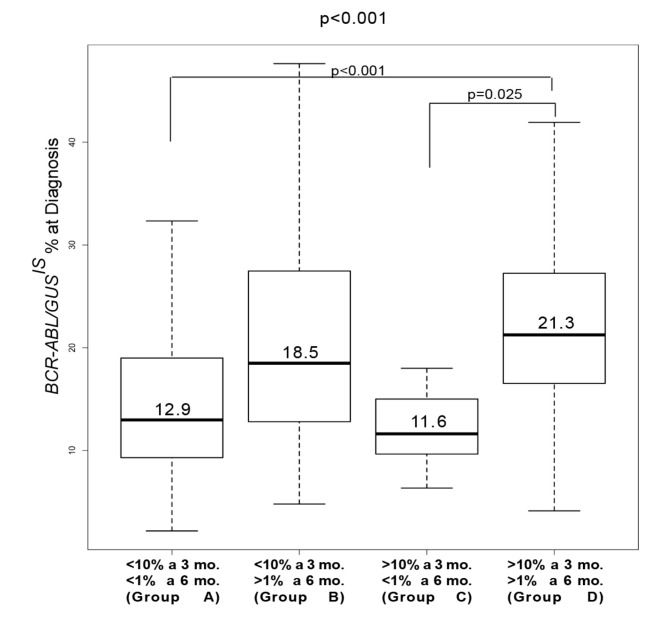
Comparison between *BCR*-*ABL1*/*GUS^IS^* levels at diagnosis and *BCR*-*ABL1/ABL1^IS^* transcripts at 3 and 6 months. *BCR*-*ABL1/GUS^IS^* levels were determined for each group and depicted as boxplots delimited by the 25th (lower) and 75th (upper) percentile. Horizontal lines above and below each boxplot indicate the 5th and 95th percentile, respectively. Thick lines in each boxplot represent median *BCR*-*ABL1/GUS^IS^* in each patients group. The *p*-value above the figure refers to statistical significance among all four groups included in the analysis while *p*-values displayed inside the figure refer to statistical significance among the population groups indicated by the bracket (comparison between groups A and D: *p* < 0.001; comparison between groups C and D: *p* = 0.025).

We and Bonecker have demonstrated that quantification of *BCR-ABL1* transcripts at diagnosis by using *GUS* rather than *ABL1* as a reference gene predicts IM response, as this measurement reflects the amount of *BCR-ABL1* transcripts within each leukemic cell [25,26]. Therefore, we explored the correlation between *BCR-ABL1/GUS^IS^* values at diagnosis and *BCR-ABL1/ABL1^IS^* levels at 3 and 6 months. We found the highest *BCR-ABL1/GUS^IS^* transcripts at diagnosis in groups B (18.5%) and D (21.3%), which were those that presented *BCR-ABL1/ABL1^IS^* values >1% at 6 months. On the contrary, both groups A and C - displaying *BCR-ABL1/ABL1^IS^* values <1% at 6 months - expressed lower *BCR-ABL1/GUS^IS^* at diagnosis (12.9% for group A and 11.6% for group C). These differences were statistically significant (*p* < 0.001; Figure 2).

### 2.4. Correlation between Risk Scores at Diagnosis and BCR-ABL1/ABL1^IS^ Transcripts at 3 and 6 Months

Multiple findings have established that low Sokal and ELTS scores are associated with better OS [10,21,22]. We calculated that in our patient cohort, the Sokal and ELTS risk categories correlated with high or low *BCR-ABL1/ABL1^IS^* transcripts at 3 and 6 months. Patients were subdivided into two categories according to their Sokal or ELTS scores (to perform a binomial analysis, low and intermediate risk were grouped together and compared to high risk) and were then stratified into the four previously described groups (A–D) according to their *BCR-ABL1/ABL1^IS^* levels at 3 and 6 months. By considering the Sokal score, we found a significant correlation in groups with concordant *BCR-ABL1/ABL1*^IS^ transcripts at 3 and 6 months (*p* < 0.02782; Table 2). Specifically, the group with the best outcome (A) presented a significantly higher percentage of low-/intermediate-risk patients as compared to the group (D) with the worst outcomes (*p* < 0.004). In groups with discordant transcripts, we found that individuals with *BCR-ABL1/ABL1^IS^* levels >1% at 6 months (group B) displayed a three-fold greater number of subjects with a high Sokal risk as compared to individuals with *BCR-ABL1/ABL1^IS^* levels >10% at 3 months (group C; 15.8% vs. 5.3%). However, these differences were not statistically significant. When we repeated this analysis considering the ELTS score, we again found a significant correlation between different risk groups and patients displaying concordant *BCR-ABL1/ABL1^IS^* transcripts at 3 and 6 months (*p* < 0.00592; Table 2). In details, more patients with low *BCR-ABL1* expression at both time points (group A) presented low/intermediate ELTS scores compared to subjects with higher *BCR-ABL1* levels at 3 and 6 months (group D; *p* = 0.0055). No significant correlation was achieved in groups with discordant *BCR-ABL1* transcripts at the two time points, possibly due to the low number of patients in each of these patient subsets.

## 3. Discussion

Quantification of *BCR-ABL1/ABL1^IS^* transcripts after 3 and 6 months of TKI therapy has become routine practice for the management of CML, as *BCR-ABL1* levels below the conventional 10% (at 3 months) and 1% (at 6 months) thresholds are associated with higher probabilities of achieving excellent failure-free survival (FFS), EFS, PFS, and OS [23,24,27]. As expected, in this report, we confirmed that patients displaying *BCR-ABL1/ABL1^IS^* transcripts below the indicated thresholds at both the 3- and 6-month landmarks presented superior outcomes compared to those with *BCR-ABL1* transcripts above the 10% and 1% values. However, we also wanted to investigate the clinical impact of *BCR-ABL1/ABL1^IS^* transcripts when the values measured at the 3- and 6-month time points were discordant: i.e., a satisfactory (<10%) value followed by an unsatisfactory one (>1%) or vice versa. A previous publication by the Hammersmith group, authored by Neelakantan and colleagues, showed that when the 3 and 6 months transcripts were discordant, the 3-month levels displayed a superior prognostic value compared to the 6-month measurement [28]. Interestingly, we found an opposite result in our patient cohort, since in subjects with discordant 3 and 6-month transcripts, the latter were predicted to be significantly more responsive to treatment compared to the former. This discrepancy might be explained by differences in the number of low (39.9% vs. 28.9%) and high (20.7% vs. 28.9%) Sokal risk patients included in our study compared to the Hammersmith paper. Indeed, a population with a less aggressive disease, as described by a low Sokal score, would probably includes individuals with oncogenic transcripts >10% at 3 months that will continue to exhibit declining transcripts over time, therefore achieving additional benefit from further treatment with the same TKI. By contrast, a population with a more aggressive disease (high Sokal score) will probably include individuals displaying *BCR*-*ABL1*/*ABL1^IS^* transcripts >10% at 3 months that have already acquired resistance to IM and are unlikely to benefit from additional treatment with the same drug. This can justify the lower predictive value of *BCR-ABL1* transcripts at the 6-month time point in the UK cohort. This hypothesis is in line with the different number of patients included in the group with high *BCR-ABL1* transcripts at 3 months but low levels at 6 months in our series (6%), compared to the UK cohort (2%). It should also be noted that our results are in agreement with those previously published by the Korean group, indicating that *BCR-ABL1* transcripts below the 1% threshold at 6 months are a reliable molecular parameter capable of identifying a subset of patients that will benefit from their assigned treatment even if their *BCR-ABL1* levels at 3 months were above the 10% value [29]. Unfortunately, we could make no comparisons between the data summarized in the UK and Korean studies and our reported findings concerning the ELTS score, as this parameter only became available in 2016 and was therefore not included in previously published manuscripts.

Both the NCCN and the ELN recommendations for the management of CML patients require disease molecular evaluations at 3, 6, and 12 months during TKI treatments. In our previous study, we demonstrated that baseline quantification of *BCR-ABL1* expression is a useful parameter to discriminate, at diagnosis, patients unlikely to benefit from standard-dose IM [25]. In the current report, we wanted to validate these data by correlating baseline *BCR-ABL1* quantification with the molecular transcripts detected at 3 and 6 months. We confirm a direct association between *BCR-ABL1* expression levels at diagnosis and IM response, as patients with *BCR-ABL1* values <1% at 6 months (groups A and C) were those expressing lower *BCR-ABL1* transcripts at diagnosis.

Finally, several clinical trials have demonstrated that patients who achieve and maintain a deep molecular response (≥MR^4^) could be considered for TKI discontinuation as they may remain in treatment-free remission (TFR), even after drug cessation. TFR is an attractive possibility because of relief from TKI toxicities, desire to plan a pregnancy, and general improvement in quality of life. Moreover, even with the advent of generic IM, TKI discontinuation may greatly relieve the financial toxicity associated with CML treatment [30]. Our data suggest that molecular responses at 3 and 6 months after IM may predict which patients will achieve greater benefit from the drug and should, therefore, be considered for treatment-free remission.

## 4. Materials and Methods

### 4.1. Patient Characteristics and Treatment

Between 1 June 2010, and 31 December 2017, a total of 184 adult patients with chronic phase (CP) CML were accrued to this study. Diagnosis of CP-CML was defined by conventional criteria. No prior treatment for CML other than hydroxyurea was allowed.

All patients received IM 400 mg daily as first-line therapy. The drug was discontinued in the presence of grade 3/4 toxicities with treatment resumed after toxicity reduction to grade 1 or complete resolution. IM responses were evaluated according to the 2013 ELN criteria [10]. Only those patients who had *BCR-ABL1* transcripts at 3 and 6 months were included in this study.

The research ethics committee (Supplementary Materials) of each recruiting institution reviewed and approved the study protocol on 10 October 2005 and all patients gave written informed consent for the data to be used in this analysis.

### 4.2. Hematologic, Cytogenetic, and Molecular Analyses Response

A complete hematological response was defined as previously reported [31]. Cytogenetic analysis was performed at diagnosis, at 3 and 6 months, and then every 6 months until a CCyR was achieved. At least 20 bone marrow cell metaphases were evaluated through conventional G-banding analysis. CCyR was defined as the failure to detect any Philadelphia chromosome (Ph)-positive metaphases in two consecutive examinations [32]. Confirmed detection of one or more Ph-positive metaphases after acquiring a CCyR was considered a cytogenetic relapse.

*BCR-ABL1* and *ABL1* expression were measured from peripheral blood (PB) samples drawn at diagnosis and then every three months using real-time PCR (qPCR) as previously described [25]. *ABL1* was used as the reference gene at any time point other than diagnosis. In addition, at diagnosis, *BCR-ABL1* expression was measured by using *GUS* as a housekeeping gene as it is a more appropriate reference gene for specimens expressing high *BCR-ABL1* [33]. All samples were processed for nucleic acid extraction in the Center of Experimental Oncology and Hematology of the A.O.U. Policlinico-Vittorio Emanuele, as previously described [34]. qPCR determinations for *BCR-ABL1/ABL1* and *BCR-ABL1/GUS* were converted to the international scale (IS), as previously reported [25]. MR^3^ was defined by *BCR-ABL1/ABL1^IS^* ≤0.1% (3-log reduction from the standardized baseline) [35,36], while MR^4^ was defined by *BCR-ABL1/ABL1^IS^* ≤0.01% (≥4-log reduction from standardized baseline) [35,36]. qPCR determinations were considered of appropriate quality only in the presence of no less than 24,000 *GUS* copies or 10,000 *ABL1* copies, as previously reported [36].

### 4.3. Statistical Analysis

Probabilities of event-free and failure-free survival, and cumulative incidence (CI) of different molecular responses, were calculated using the Kaplan–Meier method. Statistical significance between different Kaplan–Meier curves was evaluated using the Mantel–Haenszel test, as previously described [37]. Differences in *BCR-ABL1/GUS^IS^*, at diagnosis, in the four groups of patients defined according to their *BCR-ABL1/ABL1^IS^* levels at 3 and 6 months, were calculated using the ANOVA test, followed by the Tukey HSD post hoc test. Events included in our definition of EFS were death from any cause, a progression from chronic phase, IM failure according to the 2013 ELN recommendations, and development of intolerance. FFS was defined as survival without evidence of drug failure according to the latest ELN recommendations.

Differences in the occurrence of specific risk scores (Sokal or ELTS) among the four groups of patients were assessed using the Fisher exact test computing two-sided *p*-values with 95% confidence intervals. All statistical analyses were performed using R software [38].

## Figures and Tables

**Table 1:** Patient characteristics (*n* = 184).

Characteristics		%
**Follow-Up (Median mo.)**	61	
**Age (Years)**		
Median	55	
Range	20–87	
**Sex (pts n.)**		
Male	94	51.1
Female	90	48.9
**Sokal Risk Group (pts n.)**		
Low/Int	146	79.3
High	38	20.7
**ELTS Risk Group (pts n.)**		
Low/Int	163	88.6
High	21	11.4
**Transcript Type**		
e13a2 (b2a2)	74	40.2
e14a2 (b3a2)	92	50
e13a2 and e14a2	18	9.8
**Optimal Response (pts n.)**	126	68.5
**Warning (pts n.)**	10	5.4
**Intolerant (pts n.)**	9	4.9
**Failure (pts n.)**	39	21.2

ELTS: EUTOS long-term survival.

**Table 2:** Association between risk scores and *BCR-ABL1/ABL1^IS^* at 3 and 6 months.

*BCR-ABL1/ABL1^IS^*	Sokal Risk		*p*
	Low/Intermediate Risk (%)	High Risk (%)	
	*n* = 146	*n* = 38	
***<10% @ 3 mo.*** ***<1% @ 6 mo.***	98 (67.1)	18 (47.3)	0.02782
***<10% @ 3 mo.*** ***>1% @ 6 mo.***	22 (15)	6 (15.8)	
***>10% @ 3 mo.*** ***<1% @ 6 mo.***	9 (6.1)	2 (5.3)	
***>10% @ 3 mo.*** ***>1% @ 6 mo.***	17 (13.8)	12 (31.6)	
***BCR-ABL1/ABL1^IS^***	**ELTS Risk**		***p***
	**Low/Intermediate Risk**	**High Risk**	
	***n* = 163**	***n* = 21**	
***<10% @ 3 mo.*** ***<1% @ 6 mo.***	105 (64.4)	11 (52.4)	0.00592
***<10% @ 3 mo.*** ***>1% @ 6 mo.***	27 (16.6)	1 (4.8)	
***>10% @ 3 mo.*** ***<1% @ 6 mo.***	11 (6.8)	0 (0)	
***>10% @ 3 mo.*** ***>1% @ 6 mo.***	20 (12.2)	9 (42.9)	

## Supplementary Materials

The following are available online at https://www.mdpi.com/1422-0067/20/9/2226/s1.

## Abbreviations

ABL	Abelson murine leukemia
BOS	bosutinib
CCyR	complete cytogenetic response
CML	chronic myeloid leukemia
CHR	complete hematological response
DAS	dasatinib
ELN	European Leukemia Net
ELTS	EUTOS long-term survival
EFSFFS	event-free survivalfailure-free survival
GUS	β-glucuronidase
IM	imatinib mesylate
MR	molecular responses
NCCN	National Comprehensive Cancer Network
NIL	nilotinib
OR	optimal response
OS	overall survival
Ph	Philadelphia chromosome
PON	ponatinib
PFS	Progression-free survival
TKI	tyrosine kinase inhibitor
TFR	treatment-free remission
