# Supplementary material for: Clinical Implications of Discordant Early Molecular Responses in CML Patients Treated with Imatinib
Source: Int J Mol Sci. 2019 May 6;20(9):2226. doi: 10.3390/ijms20092226 (PMC6539817; doi:10.3390/ijms20092226)
Supplement: Supplementary file 1 [file ijms-20-02226-s001.pdf]

Elenco componenti Comitato Etico Catania 1

Nome e Cognome	Ruolo	Componente interno/esterno
Prof. Francesco Basile	Clinico	Interno
Dott. Vito Borzì	Clinico	Esterno
Dott.ssa Sonia Cilia	Clinico	Interno
Prof. Salvatore Di Fazzio	Clinico	Esterno
Prof. Lorenzo Malatino	Clinico	Interno
Prof. Stefano Puleo	Clinico	Interno
Prof. Sebastiano Squatrito	Clinico (Vice- Presidente)	Esterno
Dott. Gaetano Giardina	Medico di Medicina Generale	Esterno
Prof.ssa Rosaria Garozzo	Pediatra	Esterno
Dott. Antonio Gulino	Pediatra	Esterno
Prof. Filippo Palermo	Biostatistico	Esterno
Dott. Renato Scillieri	Biostatistico	Esterno
Prof. Filippo Drago	Farmacologo (Presidente)	Interno
Dott.ssa Francesca Lo Monaco	Farmacista del SSR	Interno
Dott.ssa Agata La Rosa	Farmacista del SSR	Interno
Dott.ssa Giuseppina Rizza	Farmacista del SSR	Interno
Dott. Antonio Lazzara	Direttore Sanitario	
Dott. Giuseppe Drago	Direttore Sanitario	
Dott. Salvatore Giuffrida	Direttore Sanitario	
Avv. Letterio Dario Daidone	Esperto in materia giuridica e assicurativa o un medico legale	Interno
Avv. Liliana Iachelli	Esperto in materia giuridica e assicurativa o un medico legale	Interno
Prof. Matteo Negro	Esperto in materia giuridica e assicurativa o un medico legale	Esterno
Prof. Salvatore Amato	Esperto di bioetica	Esterno
Don Antonio Sapuppo	Esperto di bioetica	Esterno
Dott.ssa Rosa Raciti	Rappresentante dell'area delle professioni sanitarie interessate alla sperimentazione	Interno
Dott. Mario Conti	Rappresentante dell'area delle professioni sanitarie interessate alla sperimentazione	Interno
Avv. Patrizia Cavallaro	Rappresentante del volontariato o dell'associazionismo di tutela dei pazienti	Esterno
Avv. Francesco Tanasi	Rappresentante del volontariato o dell'associazionismo di tutela dei pazienti	Esterno
Prof. Maurizio Di Mauro	Esperto in dispositivi medici	Esterno
Ing. Maria Luigia La Bella	In relazione all'area medico-chirurgica oggetto dell'indagine con il dispositivo medico in studio, un ingegnere clinico o altra figura professionale qualificata.	Interno
Dott. Francesco Leonardi	In relazione allo studio di prodotti alimentari sull'uomo, un esperto in nutrizione.	Esterno
Dott. Concetto Cristaudo	In relazione allo studio di nuove procedure tecniche, diagnostiche e terapeutiche, invasive e semi invasive, un esperto clinico del settore.	Interno
Prof.ssa Teresa Mattina	In relazione allo studio di genetica, un esperto in genetica.	Interno
Prof. Martino Ruggieri	In relazione allo studio di genetica, un esperto in genetica.	Interno
